# Beyond IC_50_: Reframing Microcystin Potency against Protein Phosphatase 2A by Defining Two-Step Irreversible Inhibition Kinetics

**DOI:** 10.1021/acsomega.6c03076

**Published:** 2026-04-30

**Authors:** Kelli N. Hummel, Blake B. Stringer, Sharmila I. Thenuwara, Judy A. Westrick, Jeremy J. Kodanko

**Affiliations:** † Department of Chemistry, 2954 Wayne State University, 5101 Cass Avenue, Detroit, Michigan 48202, United States; ‡ Lumigen Instrument Center, Wayne State University, 5101 Cass Avenue, Detroit, Michigan 48202, United States; § Barbara Ann Karmanos Cancer Institute, Detroit, Michigan 48201, United States

## Abstract

Microcystins (MCs) are among the most potent natural inhibitors of serine/threonine protein phosphatases, yet their toxicity is typically assessed using IC_50_ values that obscure the underlying kinetics and mechanism of enzyme inactivation. Here, we present a comprehensive kinetic analysis of protein phosphatase 2A (PP2A) inhibition by a panel of structurally diverse MC congeners, including MC-LR, MC-LA, MC-LW, MC-RR, [d-Asp^3^] MC-LR, and the noncovalent control [d-Asp^3^]­[Dhb^7^] MC-RR. Using an established PP2A inhibition assay method and modeling software to determine *K*_i_, *k*_inact_, and *k*_inact_/*K*_i_, we resolved reversible binding from covalent inactivation to characterize the two-step inhibition mechanism. All covalently competent MCs bind PP2A with picomolar affinity (*K*_i_ ≈ 3–11 pM) and undergo slow but tightly constrained covalent modification of Cys269 (*k*_inact_ ≈ 3–5 × 10^–4^ s^–1^), yielding near–diffusion-limited inactivation efficiencies (*k*_inact_/*K*_i_ ≈ 10^7^–10^8^ M^–1^ s^–1^). Structural variation among congeners primarily modulates binding equilibrium and the fraction of complexes that achieve a productive geometry for covalent bond formation, while the irreversible inactivation step remains relatively similar. Notably, [d-Asp^3^]­[Dhb^7^] MC-RR binds PP2A with picomolar affinity despite lacking a highly reactive electrophile, demonstrating that high-affinity engagement is mechanistically separable from covalent inactivation. These results reveal that MC potency is driven by exceptionally tight and persistent PP2A binding rather than fast reactivity, providing a kinetic framework that links molecular structure to sustained phosphatase inactivation and real-world toxicity during harmful algal bloom exposure.

## Graphical abstract

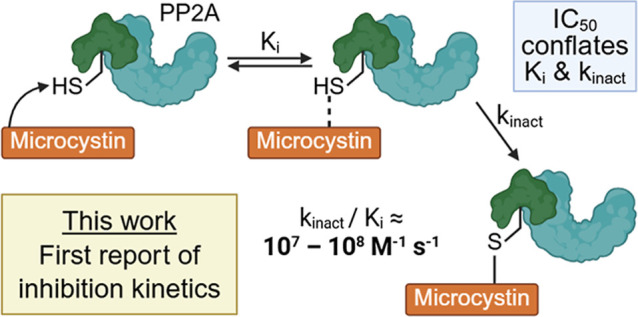

## Introduction

Cyanobacterial harmful algal blooms (cHABs) are increasingly prevalent aquatic ecological disturbances driven by eutrophication and climate, with documented expansion in frequency, duration, and geographic extent over recent decades.^1–3^ These blooms are commonly dominated by cyanobacteria, including *Microcystis*, *Planktothrix*, and *Anabaena* species, which release a diverse array of secondary metabolites, many of which are deemed toxic and collectively known as cyanotoxins.^4,5^ Among these cyanotoxins, microcystins (MCs) are a prominent family of cyclic heptapeptide hepatotoxins that pose substantial risks to environmental integrity, economic stability, and human health. MC-contaminated surface waters have been associated with fish kills, loss of biodiversity, degradation of water quality, impacting recreation, agriculture, and drinking water treatment.^6–10^ As a result, cHABs impose significant economic burdens related to water treatment infrastructure, public health impacts, and losses to fisheries and tourism.^11,12^ The accelerating global spread of cHABs has therefore become a major concern for water resource management and public health policy worldwide.

At the molecular level, one of the primary targets of MCs in animal and human cells is protein phosphatase 2A (PP2A), a highly conserved serine/threonine phosphatase that plays a central role in regulating cell cycle progression, signal transduction, apoptosis, and cytoskeletal organization.^13,14^ Together with its close homologue PP1, PP2A belongs to the phosphoprotein phosphatase (PPP) family and is responsible for maintaining phosphorylation homeostasis across numerous signaling pathways.^15^ Disruption of PPP activity leads to aberrant phosphorylation of regulatory and structural proteins, resulting in impaired signaling, cytoskeletal collapse, and cell death.^16,17^ Consistent with these molecular effects, exposure to MC-contaminated water has been linked to acute hepatotoxicity and gastrointestinal illness, as well as tumor-promoting effects in experimental models.^18^ Chronic MC exposure has further been associated with inflammation, tissue degeneration, and tumorigenesis, although epidemiological evidence in humans remains limited.^19–21^ Despite extensive biochemical and toxicological characterization of MC-mediated PP2A inhibition, the kinetic parameters governing toxin binding and irreversible enzyme inactivation have not been reported, and the implications of these kinetic features for toxicity and cellular response remain poorly understood.

MC congeners share a conserved seven–amino acid core while exhibiting complete amino acid variability, most commonly at positions 2 and 4, giving rise to a structurally diverse family comprising over 250 identified congeners.^22,23^ The heptapeptide scaffold contains highly conserved elements, most notably the hydrophobic (2S,3S,4E,6E,8S,9S)-3-amino-9-methoxy-2,6,8-trimethyl-10-phenyldeca-4,6-dienoic acid (Adda) moiety and the electrophilic *N*-methyldehydroalanine (Mdha) residue. Structural diversity arises primarily from amino acid substitutions at positions 2 and 4, with additional variation introduced through substitutions at other positions or post-translational modifications.^24^ Among these congeners, microcystin-leucine arginine (MC-LR), named for the amino acids at positions 2 and 4, is one of the most extensively studied due to its high prevalence and toxicity (Figure 1a).^25^

**Figure 1:**
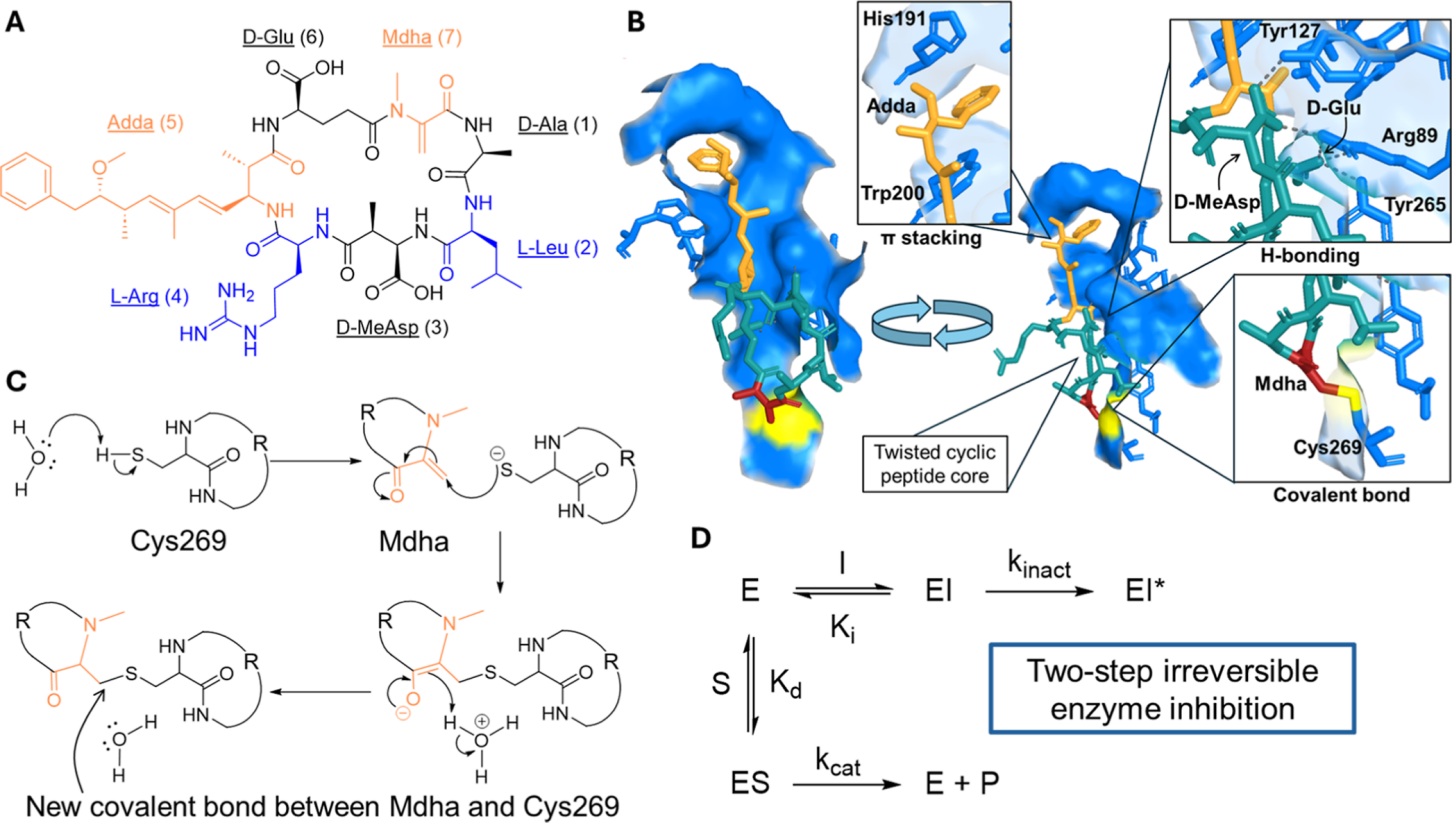
Microcystin (MC) peptide structure, its interactions with protein phosphatase 2A (PP2A) and the mechanism of inhibition. (A) The structure of MC-LR, labeled with the name of each amino acid and position number. (B) Highlighted interactions between MC-LR and PP2A, including π-stacking interactions from the position 5 Adda group, H-bonding interactions with D-MeAsp and d-Glu at positions 3 and 6, and covalent binding to the enzyme via Mdha at position 7, where cyclic peptide core is twisted, not planar. (C) Microcystins irreversibly bind to PP2A via a conjugate addition reaction between the MC Mdha residue and Cys269 of PP2A, mediated by a water molecule. R = peptide chain of MC or PP2A (D) MCs (Inhibitor, I) follow a two-step irreversible inhibition mechanism with enzyme PP2A (E). Here, PP2A can interact with a substrate (S) in equilibrium, defined by the binding affinity term *K*_d_, to form a dephosphorylated product (P) at the rate of *k*_cat_. Conversely, PP2A can interact with a MC inhibitor in equilibrium, defined by *K*_i_, before permanent inactivation occurs with the formation of a covalent bond between the MC and PP2A, measured by the rate *k*_inact_.

High-resolution structural studies have revealed that MC-LR binds within the PP2A catalytic pocket through an extensive network of noncovalent interactions, including hydrophobic contacts, π-stacking interactions, and hydrogen bonding (Figure 1b).^26,27^ Following initial binding, the electrophilic Mdha residue undergoes a conjugate addition with the thiol side chain of Cys269 in the PP2A active site, a process proposed to be facilitated by a water molecule (Figure 1c).^28^ PP2A inhibition by MC-LR therefore proceeds through a two-step irreversible mechanism, consisting of rapid, reversible enzyme–toxin association followed by slower covalent inactivation (Figure 1d). In this mechanism of inhibition, *K*_i_ (concentration units) measures the binding affinity of an inhibitor for its target enzyme at equilibrium, *k*_inact_ (time^–1^ units) is the rate of irreversible inactivation, and *k*_inact_/*K*_i_ (concentration^–1^ time^–1^ units) is the second order rate constant of inactivation describing the overall efficiency of inactivation. This mechanism locks the toxin within the active site and renders PP2A inhibition effectively irreversible on biological time scales, with important but largely unexplored consequences for toxin potency and toxicodynamic behavior.^29^

Traditionally, the potency of MCs as PP2A inhibitors has been assessed using half-maximal inhibitory concentration (IC_50_) values derived from end point enzyme activity assays. These measurements quantify the toxin concentration required to reduce PP2A activity by 50% under defined experimental conditions and have served as convenient proxies for comparing variant toxicity and developing detection assays.^30^ Using such approaches, IC_50_ values for MC inhibition of recombinant PP2A catalytic subunits have been reported in the subnanomolar range, highlighting their high apparent potency.^31^ IC_50_-based assays are widely employed in water quality monitoring and underpin regulatory frameworks defining safe levels of MC exposure.^32^ However, IC_50_ values are inherently condition-dependent and are influenced by assay duration, enzyme and substrate concentrations, and equilibrium dynamics between bound and free toxin.^33–35^ As a result, IC_50_ measurements do not provide a mechanistic description of enzyme inhibition, particularly for systems involving slow or irreversible steps that may not reach equilibrium within typical assay timeframes. Importantly, IC_50_ values are time-dependent and conflate reversible binding affinity and covalent inactivation, obscuring the distinction between thermodynamically driven association and kinetically controlled chemical modification.

Discriminating between IC_50_ values and the kinetic parameters governing two-step irreversible inhibition, therefore, requires fundamentally different assay designs.^36^ In the context of PP2A inhibition by MCs (Figure 2, top), enzymatic activity can be monitored using chromogenic substrates such as *para*-nitrophenyl phosphate (*p*-NPP) to quantify phosphatase turnover (Figure 2a). IC_50_ determination typically involves preincubation of PP2A with toxin to allow irreversible inactivation to proceed to completion prior to substrate addition, followed by measurement of residual activity (Figure 2b). In contrast, kinetic characterization requires simultaneous exposure of the enzyme to both substrate and inhibitor, with reaction progress monitored continuously over time (Figure 2c). Under these conditions, substrate turnover competes with inhibitor binding, enabling resolution of both the initial reversible association step and the subsequent rate of covalent inactivation. These distinct assay formats are therefore essential to disentangle time-dependent covalent modification from reversible binding effects and to resolve the mechanistic steps underlying irreversible PP2A inhibition.

**Figure 2:**
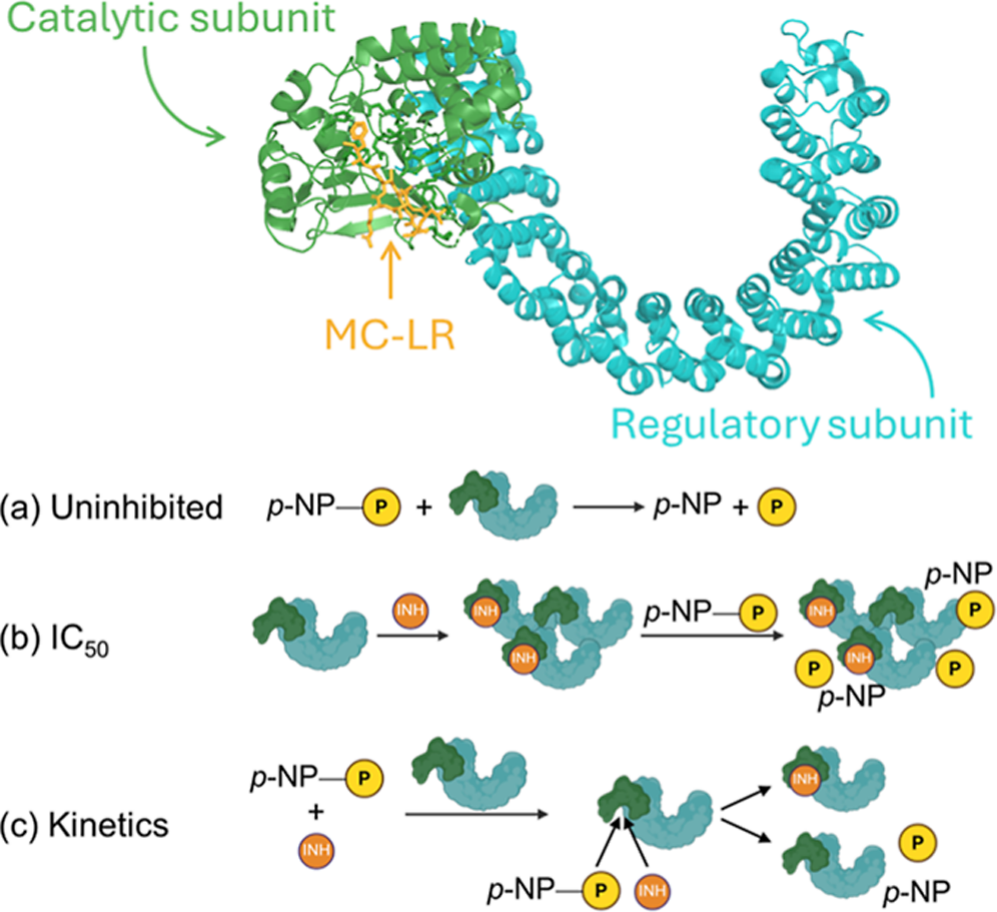
Top: color coded structure of MC-LR bound to PP2A. Bottom: schematic of the different types of reactions that can be used to measure PP2A activity. In the (a) PP2A activity assay using substrate *para*-nitrophenyl phosphate (*p*-NPP), there is a functional difference in the methods for (b) IC_50_ and (c) kinetics measurements. To measure the IC_50_ of an irreversible enzyme (b), the enzyme should be preincubated with the inhibitor to allow for the inactivation reaction to reach completion before adding the substrate and measuring activity. When measuring inhibition kinetics (c), the substrate and inhibitor are premixed followed by the addition of enzyme allowing for competition between the substrate and inhibitor over time.

A major barrier to detailed kinetic characterization of the MC–PP2A system, and other MC characterization and identification, has been the limited availability of pure and affordable toxin standards suitable for time-resolved measurements.^37–40^ Many MC congeners are difficult to isolate or synthesize in sufficient quantity, and environmental samples often contain heterogeneous mixtures that complicate quantitative analysis.^23,24^ In addition, standard PP2A inhibition assays have historically been optimized for sensitivity and throughput rather than kinetic resolution, and the slow covalent step in MC-mediated inhibition can unfold over time scales exceeding typical assay durations.^30^ Consequently, irreversible contributions to inhibition are often underestimated, and comprehensive kinetic parameters describing PP2A inactivation by MCs are largely absent from the literature.

Here, we report the first detailed kinetic characterization of PP2A inhibition by MCs, integrating real-time measurements of enzyme activity with quantitative analysis of reversible binding and covalent inactivation across representative MC variants. By resolving fundamental kinetic parameters, including the equilibrium constant for initial toxin binding (*K*_i_) and the second-order rate constant for enzyme inactivation (*k*_inact_/*K*_i_), this work distinguishes reversible and irreversible components of PP2A inhibition and places MC potency within a mechanistic framework that extends beyond traditional IC_50_ metrics. This kinetic perspective provides new insight into how MC structure influences each step of the inhibition mechanism and how these parameters relate to toxicity on biologically relevant time scales. Collectively, these findings provide foundational quantitative data necessary for improved toxicological models, regulatory assessment, and the development of more predictive monitoring tools for MC contamination in environmental and clinical contexts.

## Results and Discussion

Covalent inhibition of PP2A by MCs represents a compelling intersection of chemical reactivity and cellular regulation. MCs potently and irreversibly inhibit PP1 and PP2A through a two-step mechanism involving initial noncovalent binding followed by conjugate addition of an active-site cysteine thiol to the electrophilic Mdha residue, resulting in sustained phosphatase inactivation.^27^ Because PP2A governs essential signaling networks that control cell cycle progression, apoptosis, and stress responses, its dysregulation has profound consequences for hepatic toxicity and tumor promotion.^41^ Despite extensive structural and toxicological characterization, quantitative kinetic analysis of PP2A inactivation by distinct MC congeners remains limited. A mechanistic dissection of equilibrium binding and irreversible inactivation is therefore necessary to connect noncovalent interaction and bond formation with biological outcome. By integrating physical organic principles of covalent modification with enzyme kinetic modeling in a biologically central phosphatase system, this work clarifies how subtle structural variation among congeners can modulate both equilibrium binding and chemical reactivity, advancing a molecular-level framework for understanding MC toxicity and covalent enzyme inhibition more broadly.

To capture the key structural determinants governing PP2A inhibition while maintaining experimental tractability, a focused panel of MC congeners was selected to systematically vary features known to influence reversible binding and covalent inactivation (Figure 3). MC-LR was chosen as the reference compound because it is environmentally prevalent and well-characterized.^14,42^ Furthermore, it exhibits the canonical two-step irreversible inhibition mechanism through high-affinity binding followed by Mdha-mediated covalent modification of PP2A.^26,27^ [d-Asp^3^] MC-LR tests the impact of subtle stereochemical perturbations within the conserved core on binding geometry and covalent efficiency while preserving electrophilicity. MC-LA and MC-RR probe the effect of natural variability at positions 2 and 4 by altering charge and polarity at the binding interface without disrupting the conserved Adda and Mdha residues, enabling assessment of how side chain identity modulates equilibrium binding. MC-LW introduces increased aromatic bulk and hydrophobicity to evaluate steric and π-interactions on both binding affinity and inactivation kinetics. Finally, [d-Asp^3^]­[Dhb^7^] MC-RR serves as a mechanistic negative control, as replacement of Mdha with dehydrobutyrine (Dhb) precludes covalent bond formation,^43^ isolating the reversible binding step and validating kinetic separation of noncovalent association from irreversible inactivation.

**Figure 3:**
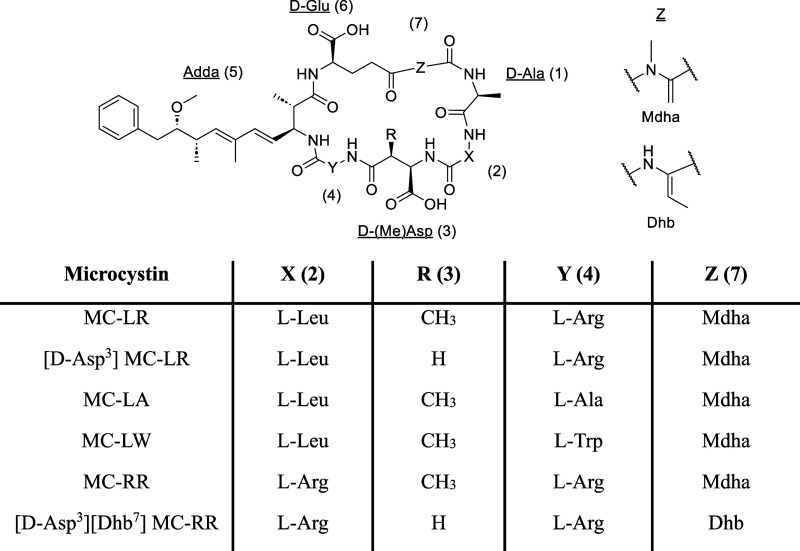
General chemical structure of MCs, structures of Mdha and Dhb at position 7 (Z), and the key structural differences between the MC congeners studied for their PP2A inhibition kinetics.

Prior to experimentation, the concentration and purity of all congeners was determined via UV–visible spectroscopy and liquid chromatography with tandem mass spectrometry (LC–MS/MS). UV detection at ∼238 nm remains widely used for MC quantification and is outlined in the International Organization for Standardization (ISO) procedure^39^ and other methods handbooks,^32^ and complementary methods such as liquid chromatography–mass spectrometry (LC–MS) are recommended to confirm analyte identity and purity.^32,39,40^ Consistent with these standard practices, vendor-reported masses were used to prepare nominal stock solutions in 100% methanol, and concentrations were corrected using molar extinction coefficients (ε) values reported in the literature.^44,45^ Because the ε for MC-LW has not been reported, the ε of MC-LR was used as an approximation. Stock concentrations were independently verified by UV–visible spectroscopy (Table S1, Figure S1) prior to PP2A inhibition assays and their purity was confirmed by LC–MS/MS (Table S2).

To measure the kinetic rate constants of enzyme inhibition described in Figure 1d, the PP2A inhibition assay was performed as previously described^47,48^ with modifications to PP2A and substrate concentration and using the order of addition shown in Figure 2c. From these data, the DynaFit enzyme modeling software was used to determine the values of *K*_i_, *k*_inact_, and *k*_inact_/*K*_i_ ^49–52^ for the MCs described above (Table 1) and representative fits are shown for each PP2A-MC pairing (Figure 4). Kinetic analysis of PP2A inhibition by five MC congeners resolved the two-step mechanism consisting of high-affinity reversible binding followed by slow covalent inactivation via conjugate addition of Cys269 to the Mdha residue. All congeners examined produced *K*_i_ values in the low-picomolar range with MC-LR being the most potent at 2.9 ± 0.4 pM and [d-Asp^3^] MC-LR the least potent Mdha-containing MC at 11.2 ± 2.3 pM while *k*_inact_ values ranged between 2.8 and 4.8 × 10^–4^ s^–1^. The narrower range of *k*_inact_ contrasts with the broader spread in *K*_i_, indicating that differences in overall inhibitory efficiency (*k*_inact_/*K*_i_) are governed more by reversible binding affinity than by the rate of covalent bond formation. This behavior is consistent with structural models in which the PP2A active site enforces a highly conserved geometry for Mdha–Cys269 bond formation once the toxin is bound in the active site.^26,27^

**Table 1:** Kinetic Parameters Determined from PP2A Inhibition Assays with the Listed MC Congeners Using the DynaFit Enzyme Model of Two-Step Irreversible Inhibition^b^

Microcystin	*K*_i_ (pM)	*k*_inact_ (×10^–4^ s^–1^)	*k*_inact_/*K*_i_ (×10^8^ M^–1^ s^–1^)	reported IC_50_ (pM)^a^
MC-LR	2.9 ± 0.4	4.8 ± 0.9	1.7 ± 0.4	32 ± 4
[d-Asp^3^] MC-LR	11.2 ± 2.3	4.5 ± 0.5	0.41 ± 0.1	---
MC-LA	5.7 ± 1	3.1 ± 0.4	0.57 ± 0.1	161 ± 2
MC-LW	7.8 ± 1	2.8 ± 0.6	0.35 ± 0.03	114 ± 3
MC-RR	7.1 ± 2	2.8 ± 0.8	0.40 ± 0.1	56 ± 2
[d-Asp^3^][Dhb^7^] MC-RR	22.8 ± 1.2	---	---	---

a Reported IC_50_ values were measured with recombinant human PP2A catalytic subunit,^31^ as was used in this study, and converted to pM for ease of comparison. [d-Asp^3^] MC-LR and [d-Asp^3^]­[Dhb^7^] MC-RR have IC_50_ values reported with PP2A isolated from rabbit skeletal muscle^46^.

b Values are expressed as the average ± standard deviation.

**Figure 4:**
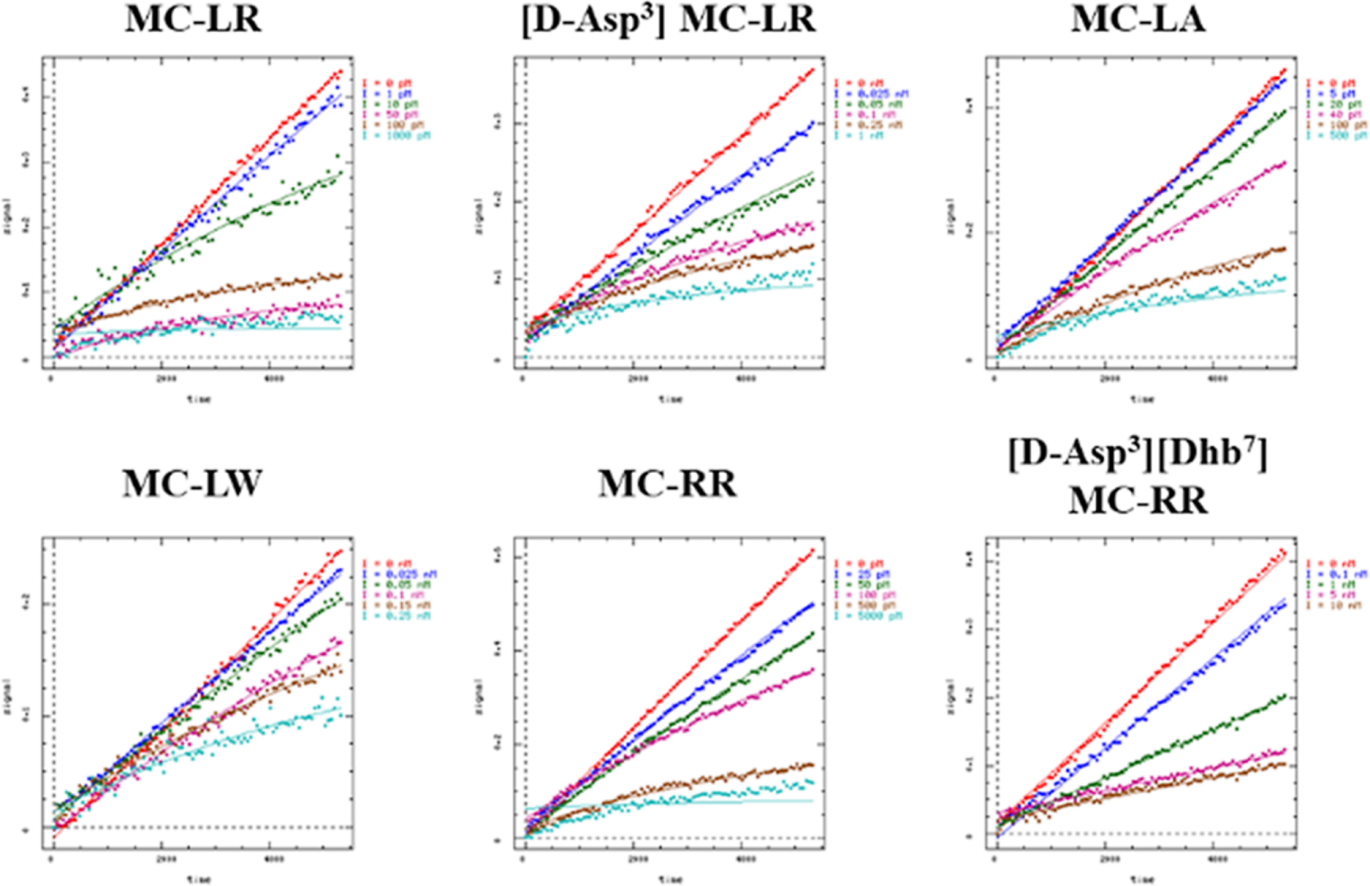
Representative fits generated from DynaFit of PP2A enzyme activity over time upon exposure of varying concentrations of the panel of MCs tested.

Among the congeners tested, MC-LR exhibited the strongest reversible binding affinity of 2.9 ± 0.4 pM and the highest *k*_inact_/*K*_i_ value of 1.7 ± 0.4 × 10^8^ M^–1^ s^–1^, establishing it as the most potent and efficient inactivator of PP2A. This finding aligns with the PP2A–MC-LR crystal structure, in which the Adda moiety occupies a hydrophobic groove and the position 4 Arg side chain engages acidic residues near the active site, together stabilizing a conformation that optimally positions the Mdha electrophile for nucleophilic attack.^26,27^ The faster *k*_inact_ observed for MC-LR further suggests that optimal binding promotes productive organization of the reactive groups and tight binding increases the residence time in the active site where Mdha and Cys269 are positioned next to each other as opposed to accelerating the chemistry through direct catalytic assistance.

Notably, omission of the β-methyl group at position 3 from MC-LR giving [d-Asp^3^]­MC-LR resulted in a ∼4-fold increase in *K*_i_ relative to MC-LR, while *k*_inact_ remained the same within error. Because this modification does not alter the charge or polarity of the side chain, the observed binding penalty is attributed to subtle steric and conformational effects rather than electrostatic interactions. Though no interactions between this side chain and PP2A are explicitly noted, the carboxylic acid of this residue forms important hydrogen bonding interactions and the effect of demethylation on PP2A toxicity appears to be dependent on other conditions.^26^ The β-methyl group in MC-LR likely contributes to preorganization of the macrocyclic peptide by restricting backbone or side chain rotamers, thereby reducing the entropic cost of adopting the bound conformation. Its removal in [d-Asp^3^]­MC-LR would increase conformational flexibility in the unbound state, leading to weaker apparent affinity despite preservation of the productive bound geometry once binding occurs. The unchanged *k*_inact_ further indicates that this methyl group is not explicitly involved in positioning the Mdha warhead for covalent reaction.

MC-LA, MC-LW, and MC-RR exhibited intermediate inhibitory efficiencies arising from effects on reversible binding and covalent inactivation. Relative to MC-LR, all three congeners showed modestly elevated *K*_i_ values accompanied by a measurable decrease in *k*_inact_, indicating that side chain variation influences the stability of the initial binding complex and the probability of achieving a covalently productive geometry. For MC-LA, removal of the Arg side chain eliminates stabilizing interactions that contribute to precise positioning of the macrocycle within the active site, resulting in both weaker binding (*K*_i_ = 5.7 ± 1 pM) and a reduced rate of Mdha–Cys269 bond formation resulting in a > 3-fold decrease in irreversible inhibition efficiency (*k*_inact_/*K*_i_ = 0.57 ± 0.1 × 10^8^ M^–1^ s^–1^) compared to MC-LR. MC-LW, with its increased hydrophobic bulk, similarly displayed a slower *k*_inact_, suggesting that the Trp side chain introduces steric constraints or altered local dynamics that subtly perturb alignment of the Mdha electrophile for attack by Cys269, thereby reducing the efficiency of the covalent step after binding has occurred. MC-RR, while binding PP2A with affinity comparable to MC-LA and MC-LW (*K*_i_ = 7.1 ± 2 pM), showed no enhancement in *k*_inact_, consistent with increased conformational heterogeneity or electrostatic frustration arising from the second Arg residue. Together, these trends indicate that altered side chain identity can decrease the fraction of bound complexes that proceed to covalent modification, reinforcing that *k*_inact_ reflects the likelihood of achieving a productive reactive pose rather than the intrinsic chemical reactivity of the Mdha warhead.

The inclusion of [d-Asp^3^]­[Dhb^7^] MC-RR provides a critical negative mechanistic control that further delineates the respective contributions of reversible binding and covalent chemistry to PP2A inhibition. The identity of [d-Asp^3^]­[Dhb^7^] MC-RR was confirmed by the thiol derivatization reaction (Figures S2,S3)^43^ revealing that the compound had been misidentified by the vendor as [d-Asp^3^] MC-RR, as previously reported.^38^ Despite lacking the Mdha electrophile required for irreversible inhibition, [d-Asp^3^]­[Dhb^7^] MC-RR binds PP2A with a *K*_i_ of 22.8 ± 1.2 pM, while the weakest compared to the congeners studied here, is still quite a potent inhibitor. This finding demonstrates that exceptionally tight binding can be achieved independently of the reactive warhead and confirms that the picomolar *K*_i_ values observed across congeners arises from conserved noncovalent interactions, particularly Adda-mediated hydrophobic engagement and macrocycle recognition. The absence of covalent inactivation by [d-Asp^3^]­[Dhb^7^] MC-RR thus establishes that the differences in *k*_inact_ observed for Mdha-containing congeners reflect variations in the fraction of bound complexes that achieve a covalently productive geometry. Together, these data highlight that reversible binding and covalent chemistry are mechanistically separable steps in PP2A inhibition and validate [d-Asp^3^]­[Dhb^7^] MC-RR as an essential control for interpreting *k*_inact_-dependent effects.

These results demonstrate that structural variation among MC congeners primarily influences the thermodynamics of PP2A engagement rather than the kinetics of covalent bond formation. The narrow range of *k*_inact_ values observed across chemically diverse congeners supports a model in which the PP2A active site imposes a conserved reactive geometry once binding occurs, while differences in side chain identity modulate the probability of achieving this productive state. This mechanistic framework provides a rationale for why MCs with similar IC_50_ values can display distinct kinetic efficiencies and underscores the importance of time-resolved kinetic analysis for dissecting structure–function relationships in covalent phosphatase inhibitors.

It is important to note that the picomolar *K*_i_ values reported here are lower than many literature IC_50_ values for MC-mediated PP2A inhibition in similar systems (Table 1),^31^ a difference that arises from both conceptual and experimental factors. IC_50_ is a composite, condition-dependent parameter that reflects enzyme concentration, incubation time, substrate competition, and the extent of irreversible inactivation achieved prior to measurement, whereas *K*_i_ isolates the equilibrium constant governing the initial reversible binding step.^33,36^ In two-step irreversible systems operating in the tight-binding regime, incomplete equilibration, substrate protection, or insufficient preincubation can inflate apparent IC_50_ values relative to the intrinsic binding affinity. Conversely, prolonged incubation under low enzyme concentrations can yield IC_50_ values that approach *K*_i_ but still conflate reversible and irreversible contributions. The differences observed here therefore do not necessarily reflect disagreement with prior potency measurements but rather underscore that IC_50_ and *K*_i_ describe distinct mechanistic quantities and are not directly interchangeable, particularly when *K*_i_ approaches or falls below the active enzyme concentration.

The kinetic regime observed for PP2A inactivation by MCs places this system at the extreme end of two-step irreversible inhibition, characterized by exceptionally tight reversible binding (*K*_i_ ≈ 3–23 pM) coupled to slow but highly constrained covalent chemistry (*k*_inact_ ≈ 3–5 × 10^–4^ s^–1^). While the absolute *k*_inact_ values are slower than those reported for cathepsins and other cysteine proteases (typically 10^–3^–10^–1^ s^–1^),^50,53–55^ the resulting *k*_inact_/*K*_i_ values approach 10^7^–10^8^ M^–1^ s^–1^, placing PP2A inactivation by MCs near the diffusion-controlled limit for enzyme–inhibitor encounters.^56^ In this regime, reversible binding becomes effectively irreversible on biological time scales, such that even modest rates of covalent bond formation are sufficient to ensure efficient enzyme inactivation. This behavior contrasts with systems that rely on stronger catalytic activation of the nucleophile, such as cathepsins, and instead reflects a binding-driven mechanism in which extensive noncovalent interactions organize the toxin within the active site, maximizing the probability of productive conjugate addition despite the relatively inactivated thiol environment of Cys269. Functionally, this kinetic architecture renders PP2A highly vulnerable to MCs: once bound, dissociation is negligible, and cumulative inactivation proceeds efficiently even with slow chemistry, providing a mechanistic explanation for the strong potency of these toxins.

For highly potent irreversible inhibitors such as MCs, reliance on IC_50_ values alone provides an incomplete and potentially misleading assessment of inhibitory potency and mechanism.^34^ IC_50_ values cannot be used as a proxy for intrinsic potency because it is a composite, time- and condition-dependent parameter that conflates reversible binding (*K*_i_) with the rate of covalent inactivation (*k*_inact_), a limitation that becomes acute when *K*_i_ values approach the picomolar regime and *k*_inact_/*K*_i_ approaches diffusion-controlled limits.^36,57,58^ As demonstrated for PP2A–MC interactions, congeners with similar IC_50_ values can differ measurably in binding affinity and inactivation efficiency, differences that are only resolved through kinetic analysis and that directly inform mechanism. Without explicit determination of *K*_i_ and *k*_inact_, structure–activity relationships risk being misinterpreted, as changes in apparent potency may reflect altered incubation time, enzyme concentration, or assay format rather than true differences in enzyme engagement.^34^ In contrast, structure–kinetic relationships (SKRs) reveal how specific structural features independently tune binding thermodynamics and covalent chemistry. Finally, because irreversible inhibition rates change over time, IC_50_ values are less predictive of downstream biological effects, including cellular persistence and toxicity, than kinetic parameters that define the rate and extent of enzyme inactivation.^57,59^ Together, these considerations show the necessity of kinetic and mechanistic characterization for interpreting, comparing, and translating the biological consequences of potent irreversible inhibitors.

In summary, direct measurement of *K*_i_, *k*_inact_, and *k*_inact_/*K*_i_ for PP2A inhibition by structurally diverse MCs reveals a kinetic regime defined by picomolar binding affinity, geometry-limited covalent chemistry, and near–diffusion-controlled inactivation efficiency. These data demonstrate that MC potency arises primarily from exceptionally tight and persistent enzyme engagement rather than from unusually fast chemical reactivity, with even subtle structural variations tuning the probability of achieving a covalently productive complex. Functionally, this kinetic architecture explains why MCs cause sustained PP2A inactivation at exceedingly low exposures, why differences among congeners can translate into distinct toxicological profiles despite similar IC_50_ values, and why recovery of phosphatase activity in exposed organisms is slow or incomplete. By resolving binding, covalent bond formation, and efficiency into discrete, quantifiable parameters, this work provides a mechanistic framework that links molecular structure to real-world outcomes, including bloom-associated toxicity, risk assessment, and the design of mitigation strategies that must account for the essentially irreversible nature of PP2A inhibition in biological systems.

## Methods

### PP2A Inhibition Assay

The PP2A inhibition assay was performed as previously described^47,48^ with modifications using a Tecan Spark Multimode Microplate Reader. A full list of materials can be found in Table S3. Purified PP2A catalytic subunit was diluted in enzyme solution without dithiothreitol (DTT), aliquoted for single use (including a blank, control, and five concentrations of inhibitor in triplicate), snap frozen in liquid nitrogen, and stored at −80 °C. Solutions of 10 mM MnCl_2_ and 5 mg/mL BSA were prepared, aliquoted, and stored at −20 °C for future use. Buffer solutions, 0.2 mM MgCl_2_, 1 mM EGTA were prepared at stored at 4 °C. All other solutions were prepared or mixed fresh daily. Reaction buffer, substrate solution, and enzyme solution were prepared fresh daily as described. The final concentration of PP2A was modified to 0.5 U/mL and the initial concentration of *p*-nitrophenyl phosphate (*p*-NPP) was modified to 30 mM. Each reaction was performed in triplicate. Prewarmed substrate solution (200 μL) and MC in reaction buffer (20 μL) were added to each well of a 96-well plate. The plate was placed in the 37 °C plate reader while the PP2A enzyme was reconstituted in enzyme solution. The PP2A solution was added to empty adjacent wells in the 96-well plate and incubated for 5 min at 37 °C for controlled activation of the PP2A enzyme. After preincubation, the PP2A solution (20 μL) was added to wells containing the substrate solution and MC. A set of triplicate wells was designated the blank, containing only substrate solution, reaction buffer, and enzyme solution without PP2A. Another set of triplicate wells was designated the control, containing substrate solution, reaction buffer, and PP2A in enzyme solution. Once mixed to give a total reaction volume of 240 μL, the plate was placed in the 37 °C plate reader and absorbance at 405 nm was recorded every minute for 1.5 h.

### Progress Curves of PP2A Inhibition by MCs Using DynaFit

Modeling of two-step irreversible inhibition and equilibrium kinetics using DynaFit were performed as previously described.^49–52^ After absorbance data was collected for PP2A inhibition, raw data were fit to the two-step irreversible inhibition model presented in Figure 1d to determine *K*_i_, *k*_inact_, and *k*_inact_/*K*_i_ or to an equilibrium binding model in the case of [d-Asp^3^]­[Dhb^7^] MC-RR to determine *K*_i_. Example scripts used for these fits are presented in Figures S4,S5. Representative fits for each PP2A/MC reaction are shown in the main text; the individual fits used to calculate the average of the indicated kinetic parameters are shown in Figures S6–S10.

## Supplementary Material



## Notes

Detailed experimental procedures, supplementary figures, and tables, including MC purity and UV–visible spectra, identify confirmation of [d-Asp^3^]­[Dhb^7^] MC-RR, example scripts used in the DynaFit modeling software, and individual fits used to calculate average kinetic values (PDF)

## Abbreviations

Adda: (2S,3S,4E,6E,8S,9S)-3-amino-9-methoxy-2,6,8-trimethyl-10-phenyldeca-4,6-dienoic acid
cHABs: cyanobacterial harmful algal blooms
Dhb: dehydrobutyrine
IC_50_: half-maximal inhibitory concentration
ISO: International Organization for Standardization
*K*_i_: equilibrium constant enzyme–inhibitor binding
*k*_inact_: rate constant of irreversible inhibition
*k*_inact_/*K*_i_: second-order rate constant of enzyme inactivation
LC-MS: liquid chromatography–mass spectrometry
LC-MS/MS: liquid chromatography with tandem mass spectrometry
*p*-NPP: *para*-nitrophenyl phosphate
MC: microcystin
MC-LR: microcystin-leucine arginine
Mdha: *N*-methyldehydroalanine
PP2A: protein phosphatase 2A
PPP: phosphoprotein phosphatase
SKR: structure–kinetic relationship
