# Supplementary material for: Beyond IC50: Reframing Microcystin Potency against Protein Phosphatase 2A by Defining Two-Step Irreversible Inhibition Kinetics
Source: ACS Omega. 2026 Apr 30;11(19):29202–10. doi: 10.1021/acsomega.6c03076 (PMC13191494; doi:10.1021/acsomega.6c03076)
Supplement: Supplementary file 1 [file ao6c03076_si_001.pdf]

Supporting Information

Beyond IC_{50}: Reframing microcystin potency against protein phosphatase 2A by defining two-step irreversible inhibition kinetics

Kelli N. Hummel,[†] Blake B. Stringer,[†] Sharmila I. Thenuwara,[‡] Judy A. Westrick,[†] Jeremy J. Kodanko[†§*]

[†]*Department of Chemistry, Wayne State University, 5101 Cass Avenue, Detroit, Michigan 48202, United States*

[‡]*Lumigen Instrument Center, Wayne State University, 5101 Cass Avenue, Detroit, Michigan 48202, United States*

[§]*Barbara Ann Karmanos Cancer Institute, Detroit, Michigan 48201, United States*

Table of Contents

Table S1: Determination of MC stock concentration by UV-Visible spectroscopy……………S2

Figure S1: UV-visible spectra of MC congeners…………………………………………..S3

Table S2: Tabulated purity of MC congeners by LC-MS/MS……………………………………S4

Figures S2-3: Chemical derivatization of MC-RR and [D-Asp^3][Dhb^7] MC-RR……..……..S5-6

Table S3: Detailed table of reagents used in PP2A inhibition assay……………………………..S7

Figures S4-5: Example scripts used in DynaFit to model two-step irreversible inhibition and equilibrium binding…………………………………………………………………..…S8-9

Figures S6-10: Individual fits produced from DynaFit for the determination of binding and rate constants……………………………………………………………………………S10-13

References……………………………………………………………………….....…..S14

Instrumentation

The electronic absorption spectra were collected using a Cary 60 spectrophotometer or a Molecular Devices SpectraMax M2 spectrometer using 1 cm × 1 cm quartz cuvettes.

Absorbance data in enzyme activity experiments were collected using a Tecan Spark Multimode Microplate Reader.

Analysis of MC purity was performed using a Thermo Scientific Ultimate 3000 LC system attached to a Thermo Scientific Orbitrap Exploris 120 High-Resolution Mass Spectrometer containing a Restek Raptor Biphenyl 2.7 µm x 100 mm x 2.1 mm column.

Analysis of MC stock concentration via UV-visible spectroscopy

MC standards were purchased from Enzo and dissolved in LCMS-grade methanol to make nominal 100 ppm or 25 ppm stock solutions based on the purchased mass. The stock solutions were diluted to working concentrations of 5-20 ppm in a volume of 300 µL. The UV-visible spectrum of each MC was taken and corrected against a methanol blank. The Beer-Lambert Law equation was used to calculate the concentration of solutions using their given molar extinction coefficient (ε) (Table S1) and multiplied by the given dilution factor to determine the concentration of each stock solution. The extinction coefficients of MC-LR,[S1] [D-Asp^3] MC-LR,[S2] MC-LA,[S3] MC-RR,[S1] and [D-Asp^3][Dhb^7] MC-RR[S4] are reported. As the extinction coefficient of MC-LW is unreported, the extinction coefficient of MC-LR was used to approximate the concentration. These data are presented in ppm (green) and µM (blue) concentrations, comparing nominal and calculated concentrations, in Table S1 and the spectra are shown in Figure S1.

Congener	Nominal [Stock] (ppm)	Nominal [Stock] (µM)	Abs (λ_{max} nm)	ε (M^{-1} cm^{-1})	Calculated [Stock] (ppm)	Calculated [Stock]* (µM)
**MC-LR**	100	100.5	0.43 (238)	39800	107.5	108.0
**[D-Asp^3] MC-LR**	25	25.5	0.19 (238)	31600	29.5	30.1
**MC-LA**	100	109.9	0.76 (238)	36500	94.7	104.1
**MC-LW**	25	24.4	0.37 (238)	Not reported	23.8	23.2[†]
**MC-RR**	100	96.3	0.78 (238)	39800	101.7	98.0
**[D-Asp^3][Dhb^7] MC-RR**	100	97.6	0.47 (239)	50400[‡]	95.5	93.3

*Calculated using the Beer-Lambert Law equation where ε (M^{-1} cm^{-1}) = 39800 (MC-LR),[†] 31600 ([D-Asp^3] MC-LR), 36500 (MC-LA), 39800 (MC-RR), 50400 ([D-Asp^3][Dhb^7] MC-RR).

[‡]The ε for [D-Asp^3][Dhb^7] MC-RR is considerably higher than the other MCs but was used since it has been measured and reported.

Table S1. Determination of the concentration of MC stock solutions using UV-Visible absorbance measurements and reported molar extinction coefficients (ε) where reported.

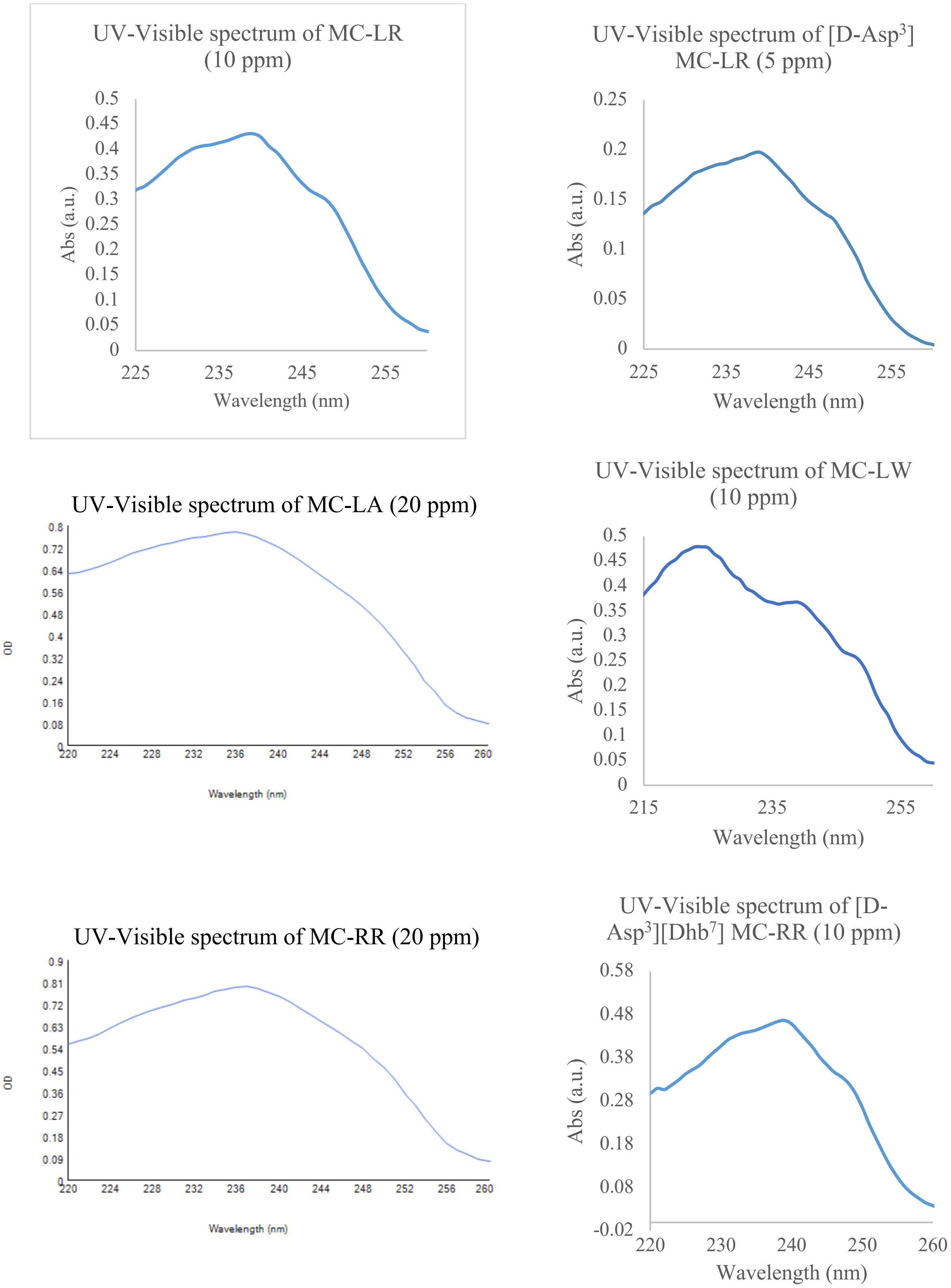

Figure S1. UV-Visible spectra of the MC congeners evaluated in this study at the indicated working concentration. Spectral features of the MCs appear between 215-260 nm.

Determination of MC purity by LC-MS/MS

All MC standards were purchased from Enzo. Each microcystin standard was brought to a final concentration of 25 ppm in methanol. These solutions were then loaded onto a Thermo Scientific Ultimate 3000 LC system. This system was attached to a Thermo Scientific Orbitrap Exploris 120 High-Resolution Mass Spectrometer. For each MC solution, 10 µL was injected and carried onto a Restek Raptor Biphenyl 2.7 µm x 100 mm x 2.1 mm column via water (A) and acetonitrile (B) both with 0.1% formic acid at 0.5 mL/min. The following gradient was applied: 0-1 min, 5-5% B; 1-11 min, 25-50% B; 11-13 min, 50-100% B; 13-15 min, 100-100% B; 15-15.1 min, 100-5% B; 15.1-20 min, 5-5% B. The MS scan range was from 120-1800 m/z. The RF lens was set to 70%. Both positive (+2800 V) and negative (−2500V) mode were analyzed. The resolution was set to 15,000 with the higher energy collision dissociation set to 30%. Microcystin contaminants of each standard were quantified on the assumption that the standards and contaminants ionized similarly and are listed in Table S2.

Tested Standard (Brand/Lot #)	Putative Contaminants	Contaminant *m/z*	Signal of Contaminant/ Standard (%)	Confirmed Contaminant MS^2 Fragments *m/z*
MC-LR (Enzo/L30814)	D-Asp^3 MC-LR<br>NMe-Ser^7 MC-LR	981.5395<br>1013.5654	0.89<br>1.02	127.0862, 135.0804, 213.0874<br>70.0650, 107.0853, 135.0803
MC-LA (Enzo/30492)	MC-HilA<br>MC-LA Salt	924.5059<br>955.5500	0.89<br>1.52	107.0853, 135.0803, 227.1026<br>107.0853, 127.0862, 135.0803
MC-RR (Enzo/L30743)	None	N/A	N/A	N/A
MC-LW (Enzo/L30836)	D-Asp^3 MC-LF	972.5073	2.3	135.0802, 249.1229, 361.1752
[D-Asp^3] MC-LR (Enzo/L30788)	MC-LR<br>NMe-Ser^7 MC-LR	995.5526<br>1013.5642	2.11<br>0.72	70.0649, 107.0851, 135.0801<br>70.0649, 86.0961, 135.0800
[D-Asp^3][Dhb^7] MC-RR* (Enzo/L30235)	Gly^1-D-Asp^3 MC-RR	1010.5400	1.19	70.0649, 107.0855, 135.0802

*Labeled by Enzo as [D-Asp^3] MC-RR

Table S2. Tabulated purities of the MC congeners evaluated in this study, as analyzed by LC-MS/MS.

Confirmation of [D-Asp^3][Dhb^7] MC-RR via thiol derivatization

The procedure for chemical derivatization of MCs[S5] to distinguish between Mdha and Dhb residues was performed as previously reported.[S6] The electrophilic Mdha reactions with a thiol, whereas the Dhb reacts minimally. To confirm the identity of [D-Asp^3][Dhb^7] MC-RR, both the putative [D-Asp^3][Dhb^7] MC-RR and MC-RR were reacted with 2-mercaptoethanol. Samples were analyzed using a TSQ Altis LC-MS/MS system, as previously described.[S5,6] Chromatograms of the MC-RR samples and [D-Asp^3][Dhb^7] MC-RR samples are shown in Figures S2-3.

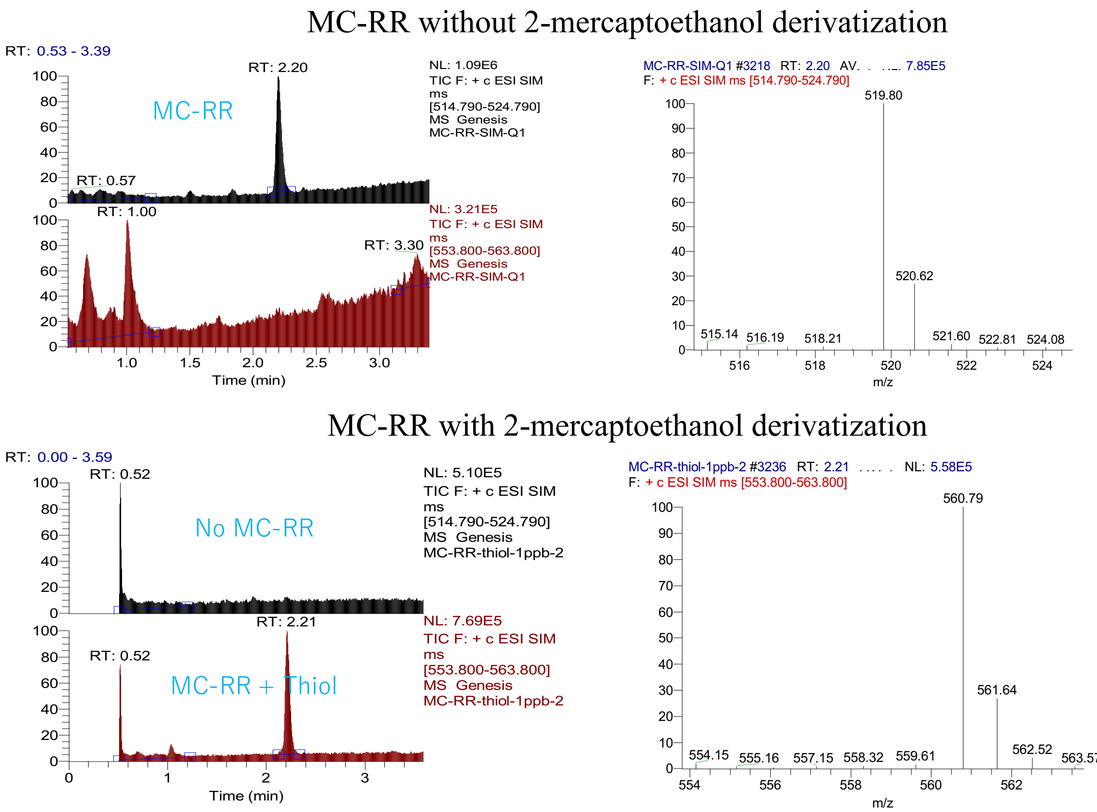

Figure S2. Chemical derivatization of MC-RR indicating the formation of an MC-RR—thiol bond.

[D-Asp^{3}][Dhb^{7}] MC-RR without 2-mercaptoethanol derivatization

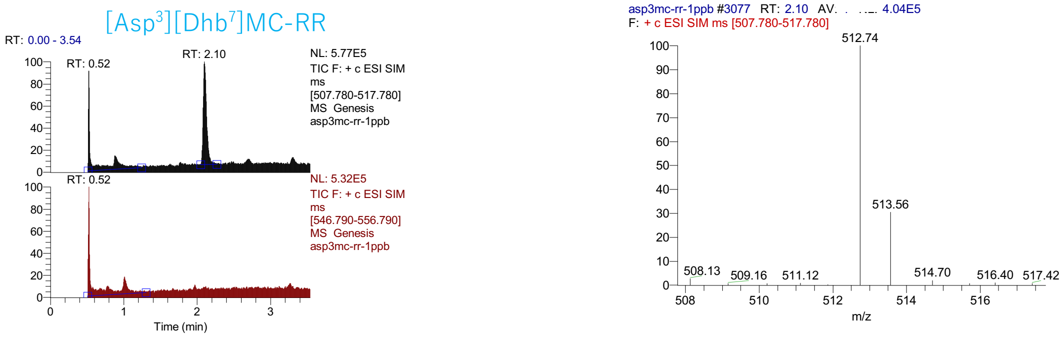

[D-Asp^{3}][Dhb^{7}] MC-RR with 2-mercaptoethanol derivatization

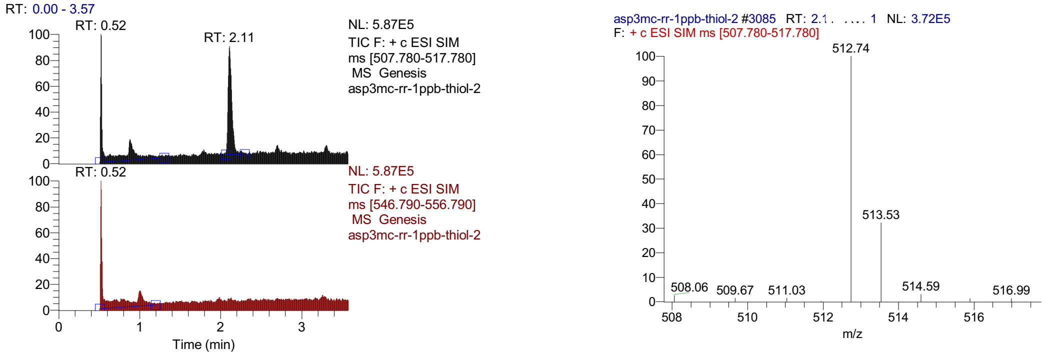

Figure S3. Chemical derivatization of [D-Asp^{3}][Dhb^{7}] MC-RR indicating that there is little to no bond formation between the [D-Asp^{3}][Dhb^{7}] MC-RR (labeled as [D-Asp^{3}] MC-RR) and 2-mercaptoethanol.

PP2A inhibition assay

All solutions used in the PP2A inhibition assay were prepared as previously described,[S7] with the exception of the initial concentrations of *p*-NPP and PP2A, which were adjusted to 30 mM and 6 U/mL, respectively.

Reagent/Material	CAS No.	Vendor	Catalog No.	Lot No.	Comments
Tris(hydroxymethyl)aminomethane	77-86-1	Fisher BioReagents	BP152-500	161603	White solid; formed colorless solution.
Dithiothreitol (DTT)	3483-12-3	Thermo Scientific	R0861	3199059	Assembling lot no. 3230309. White solid; formed colorless solution.
Manganese (II) chloride	7773-01-5	Alfa Aesar	11868	W06C015	Pale white-pink flakes; formed colorless solution.
Bovine serum albumin (BSA)	9048-46-8	Sigma Aldrich	A7030-10G	424204	Off-white powder; formed colorless solution.
Ethylene glycol bis(2-aminoethyl)tetraacetic acid (egtazic acid, EGTA)	67-42-5	Sigma Aldrich	E3889-10G	450205	Source No. 399378. White solid; formed colorless solution.
Magnesium (II) chloride	7786-30-3	EMD Millipore	442611-500GM	4289695	White solid; formed colorless solution.
p-Nitrophenyl phosphate (*p*-NPP)	4264-83-9	Sigma Aldrich	N4645-1G	447199	Pale yellow solid; formed pale yellow solution.
				496178	White solid; formed colorless solution.
Sodium acetate	127-09-3	Sigma Aldrich	S2889-250G	395711	Source No. 373117. White powder; formed colorless solution,
Protein Phosphatase 2A C subunit (L309 deletion mutant; human, recombinant)	NA	Cayman Chemical	10011237	804287-1	2 units of the same batch were purchased separately.
				804287-1	

Table S3. Detailed list of reagents used in the PP2A inhibition assay experiments.

Modeling PP2A inhibition kinetics in DynaFit

As previously described,[S8–11] raw absorbance data over time for each PP2A inhibition assay were used in the DynaFit software (BioKin Ltd., Version 4.11.114) to determine the binding and rate constants of two-step irreversible inhibition (Figure S4) and equilibrium inhibition (Figure S5). The individual fits of experiments used to calculate the average of the reported kinetic parameters are shown in Figures S6-10.

```
all concentrations are expressed in uM
[task]
   data = progress
   task = fit
   model = two step irreversible inhibition ?
[mechanism]
   E + S <==> E.S      :    ka.S  kd.S
   E.S ---> E + P      :    kcat
   E + I <==> E.I      :    ka.I  kd.I
   E.I ---> E-I        :    k.inact
[constants]
   ka.S = 30 ?, kd.S  = 25000 ?                    ; ka.S in uM-1 s-1, kd.S in s-1
   kcat = 100 ?                                    ; kcat in s-1
   ka.I = 10000000 ?, kd.I = 30 ?                  ; ka.I in uM-1 s-1, kd.I in s-1
   k.inact = 0.001 ?  (0.0001 .. 0.01)             ; kinact in s-1
[concentrations]
   E = 0.0000001 ?                                  ; enzyme in uM
   S = 10000.0 ?                                   ; substrate in uM, 10 mM
[responses]
   P = 100 ?
[data]
   directory  ./csv
   sheet      xxx.csv
   column 2 | conc I = 0.000                                  | offset = auto ? |
label = I = 0 pM
   column 3 | conc I = 0.0000025 ? (0.000002 .. 0.000003)     | offset = auto ? |
label = I = 2.5 pM
   column 4 | conc I = 0.000005 ? (0.0000045 .. 0.0000055)    | offset = auto ? |
label = I = 5 pM
   column 5 | conc I = 0.00001 ? (0.000005 .. 0.000015)       | offset = auto ? |
label = I = 10 pM
   column 6 | conc I = 0.00002 ? (0.000015 .. 0.000025)       | offset = auto ? |
label = I = 20 pM
   column 7 | conc I = 0.00005 ? (0.000045 .. 0.000055)       | offset = auto ? |
label = I = 50 pM
[settings] {Constraints} | Concentrations = 0.01
[output]
   directory  ./Outputs/XXX
[end]
```

Figure S4. Example script used in DynaFit to model two-step irreversible inhibition of PP2A by Mdha-containing MCs.

```
all concentrations are expressed in uM
[task]
   data = progress
   task = fit
   model = equilibrium ?
[mechanism]
   E + S <==> E.S      :    ka.S  kd.S
   E.S ---> E + P      :    kcat
   E + I <==> E.I      :    ka.I  kd.I
[constants]
   ka.S = 30 ?, kd.S  = 25000 ?                        ; ka.S in uM-1 s-1, kd.S in s-1
   kcat = 100 ?                                         ; kcat in s-1
   ka.I = 10000000 ?, kd.I = 200 ?                      ; ka.I in uM-1 s-1, kd.I in s-1
[concentrations]
   E = 0.0000001 ?                                      ; enzyme in uM
   S = 10000.0 ?                                        ; substrate in uM, 10 mM
[responses]
   P = 100 ?
[data]
   directory  ./csv
   sheet      xxx.csv
   column 2 | conc I = 0.000                                      | offset = auto ? |
label = I = 0 nM
   column 3 | conc I = 0.0001 ? (0.00005 .. 0.00015)             | offset = auto ? |
label = I = 0.1 nM
   column 4 | conc I = 0.00025 ? (0.0002 .. 0.0003)              | offset = auto ? |
label = I = 0.25 nM
   column 5 | conc I = 0.0005 ? (0.00045 .. 0.00055)             | offset = auto ? |
label = I = 0.5 nM
   column 6 | conc I = 0.001 ? (0.0005 .. 0.0015)                | offset = auto ? |
label = I = 1 nM

[settings] {Constraints} | Concentrations = 0.01
[output]
   directory  ./Outputs/XXX
[end]
```

Figure S5. Example script used in DynaFit to model equilibrium binding of [D-Asp^3][Dhb^7] MC-RR with PP2A.

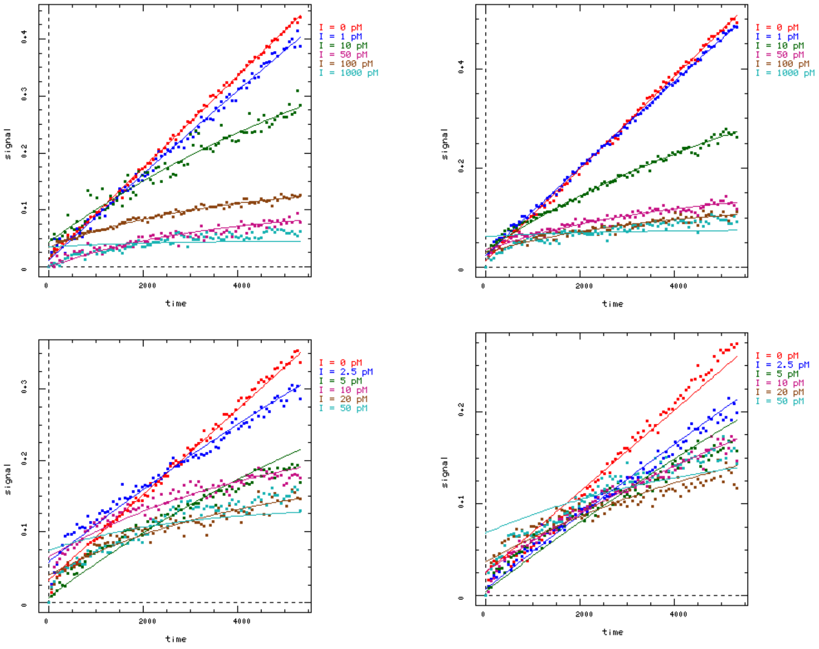

Figure S6. Individual fits produced through modeling in DynaFit to determine the average of the kinetic constants of two-step irreversible inhibition of PP2A by MC-LR.

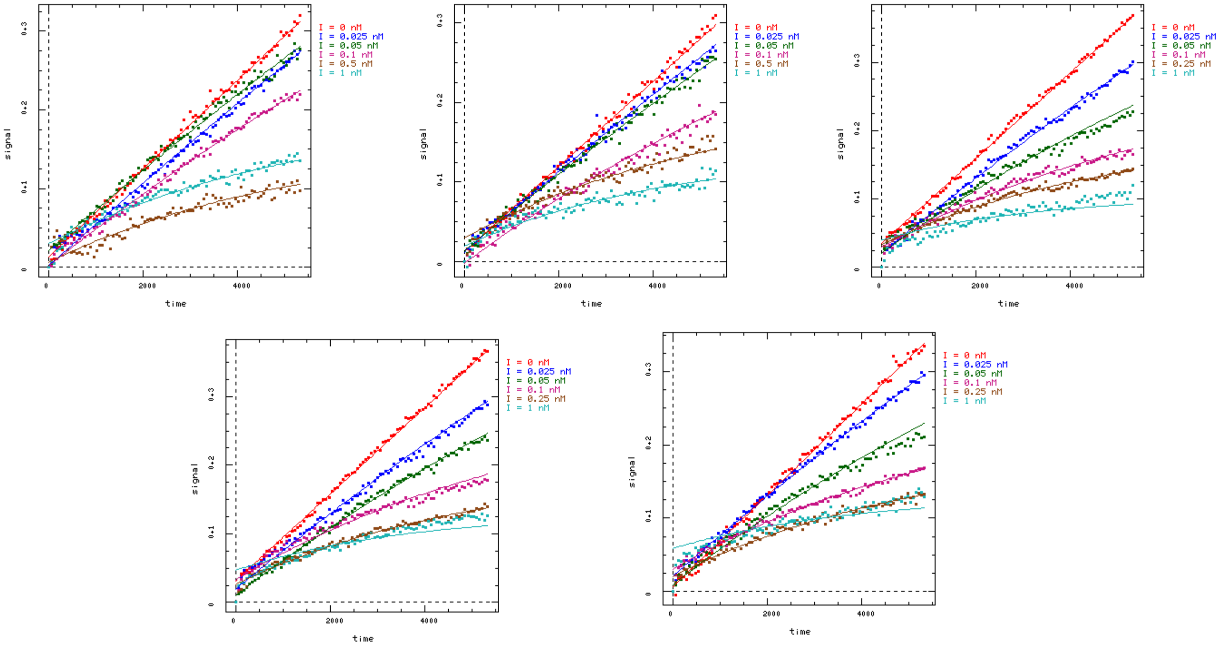

Figure S7. Individual fits produced through modeling in DynaFit to determine the average of the kinetic constants of two-step irreversible inhibition of PP2A by [D-Asp^3] MC-LR.

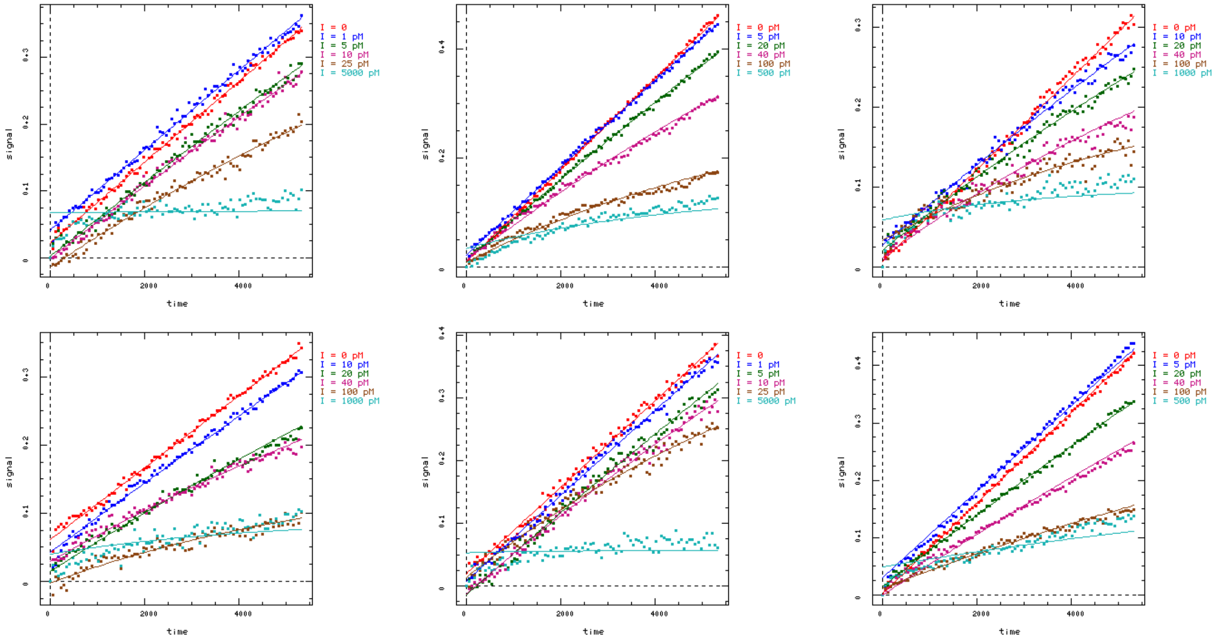

Figure S7. Individual fits produced through modeling in DynaFit to determine the average of the kinetic constants of two-step irreversible inhibition of PP2A by MC-LA.

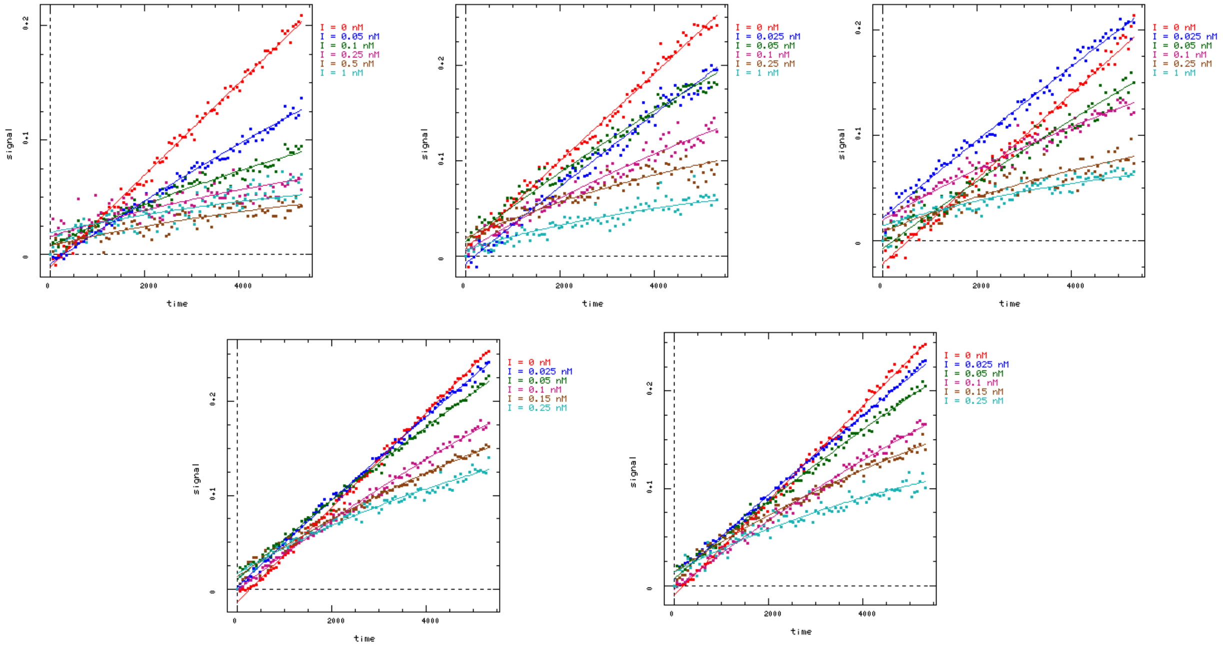

Figure S8. Individual fits produced through modeling in DynaFit to determine the average of the kinetic constants of two-step irreversible inhibition of PP2A by MC-LW.

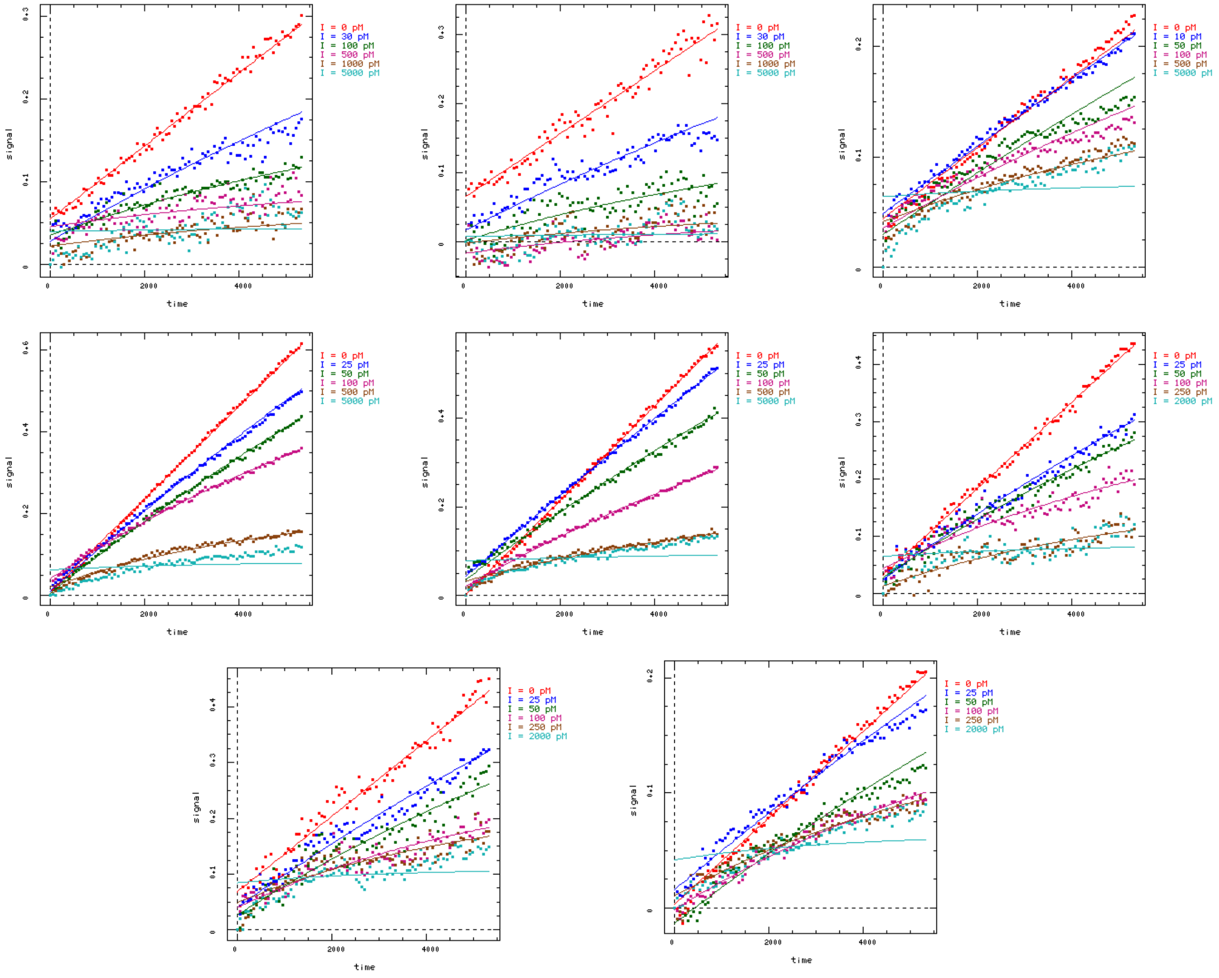

Figure S9. Individual fits produced through modeling in DynaFit to determine the average of the kinetic constants of two-step irreversible inhibition of PP2A by MC-RR.

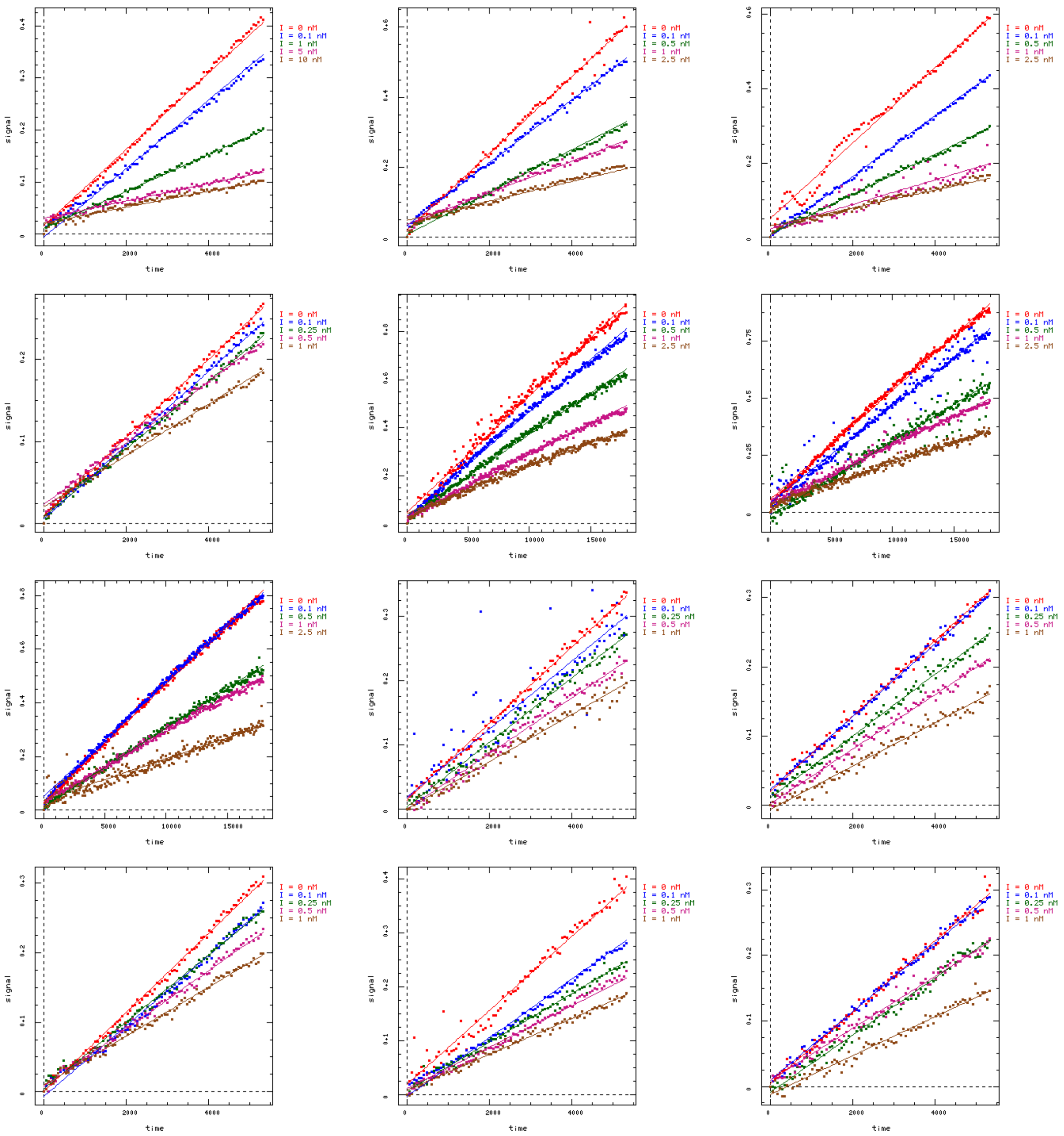

Figure S10. Individual fits produced through modeling in DynaFit the equilibrium binding constant (K_i) of [D-Asp^3][Dhb^7] MC-RR with PP2A.

References

S1. Harada, K.; Matsuura, K.; Suzuki, M.; Watanabe, M. F.; Oishi, S.; Dahlem, A. M.; Beasley, V. R.; Carmichael, W. W. Isolation and Characterization of the Minor Components Associated with Microcystins LR and RR in the Cyanobacterium (Blue-Green Algae). *Toxicon* **1990**, *28* (1), 55–64. https://doi.org/10.1016/0041-0101(90)90006-S.

S2. Harada, K.; Ogawa, K.; Matsuura, K.; Nagai, H.; Murata, H.; Suzuki, M.; Itezono, Y.; Nakayama, N.; Shirai, M.; Nakano, M. Isolation of Two Toxic Heptapeptide Microcystins from an Axenic Strain of Microcystis Aeruginosa, K-139. *Toxicon* **1991**, *29* (4–5), 479–489. https://doi.org/10.1016/0041-0101(91)90022-J.

S3. Backer, L. C.; McNeel, S. V.; Barber, T.; Kirkpatrick, B.; Williams, C.; Irvin, M.; Zhou, Y.; Johnson, T. B.; Nierenberg, K.; Aubel, M.; LePrell, R.; Chapman, A.; Foss, A.; Corum, S.; Hill, V. R.; Kieszak, S. M.; Cheng, Y.-S. Recreational Exposure to Microcystins during Algal Blooms in Two California Lakes. *Toxicon* **2010**, *55* (5), 909–921. https://doi.org/10.1016/j.toxicon.2009.07.006.

S4. Blom, J. F.; Robinson, J. A.; Jüttner, F. High Grazer Toxicity of [D-Asp3,(E)-Dhb7]Microcystin-RR of Planktothrix Rubescens as Compared to Different Microcystins. *Toxicon* **2001**, *39* (12), 1923–1932. https://doi.org/10.1016/S0041-0101(01)00178-7.

S5. Miles, C. O.; Sandvik, M.; Haande, S.; Nonga, H.; Ballot, A. LC-MS Analysis with Thiol Derivatization to Differentiate [Dhb^7]- from [$Mdha^7$]-Microcystins: Analysis of Cyanobacterial Blooms, *Planktothrix* Cultures and European Crayfish from Lake Steinsfjorden, Norway. *Environ. Sci. Technol.* **2013**, *47* (9), 4080–4087. https://doi.org/10.1021/es305202p.

S6. Birbeck, J. A.; Peraino, N. J.; O'Neill, G. M.; Coady, J.; Westrick, J. A. Dhb Microcystins Discovered in USA Using an Online Concentration LC–MS/MS Platform. *Toxins* **2019**, *11* (11), 653. https://doi.org/10.3390/toxins11110653.

S7. Heresztyn, T. Determination of Cyanobacterial Hepatotoxins Directly in Water Using a Protein Phosphatase Inhibition Assay. *Water Res.* **2001**, *35* (13), 3049–3056. https://doi.org/10.1016/S0043-1354(01)00018-5.

S8. Denison, M.; Garcia, S. P.; Ullrich, A.; Podgorski, I.; Gibson, H.; Turro, C.; Kodanko, J. J. Ruthenium-Cathepsin Inhibitor Conjugates for Green Light-Activated Photodynamic Therapy and Photochemotherapy. *Inorg. Chem.* **2024**, *63* (17), 7973–7983. https://doi.org/10.1021/acs.inorgchem.4c01008.

S9. Arora, K.; Herroon, M.; Al-Afyouni, M. H.; Toupin, N. P.; Rohrabaugh, T. N.; Loftus, L. M.; Podgorski, I.; Turro, C.; Kodanko, J. J. Catch and Release Photosensitizers: Combining Dual-Action Ruthenium Complexes with Protease Inactivation for Targeting Invasive Cancers. *J. Am. Chem. Soc.* **2018**, *140* (43), 14367–14380. https://doi.org/10.1021/jacs.8b08853.

S10. Huisman, M.; White, J. K.; Lewalski, V. G.; Podgorski, I.; Turro, C.; Kodanko, J. J. Caging the Uncageable: Using Metal Complex Release for Photochemical Control over Irreversible Inhibition. *Chem. Commun.* **2016**, *52* (85), 12590–12593. https://doi.org/10.1039/C6CC07083C.

S11. Kuzmič, P. Program DYNAFIT for the Analysis of Enzyme Kinetic Data: Application to HIV Proteinase. *Anal. Biochem.* **1996**, *237* (2), 260–273. https://doi.org/10.1006/abio.1996.0238.
